# Supplementary figures and images for: The Effect of Treatment-Induced Viral Eradication on Cytokine and Growth Factor Expression in Chronic Hepatitis C
Source: Viruses. 2022 Jul 24;14(8):1613. doi: 10.3390/v14081613 (PMC9394470; doi:10.3390/v14081613)

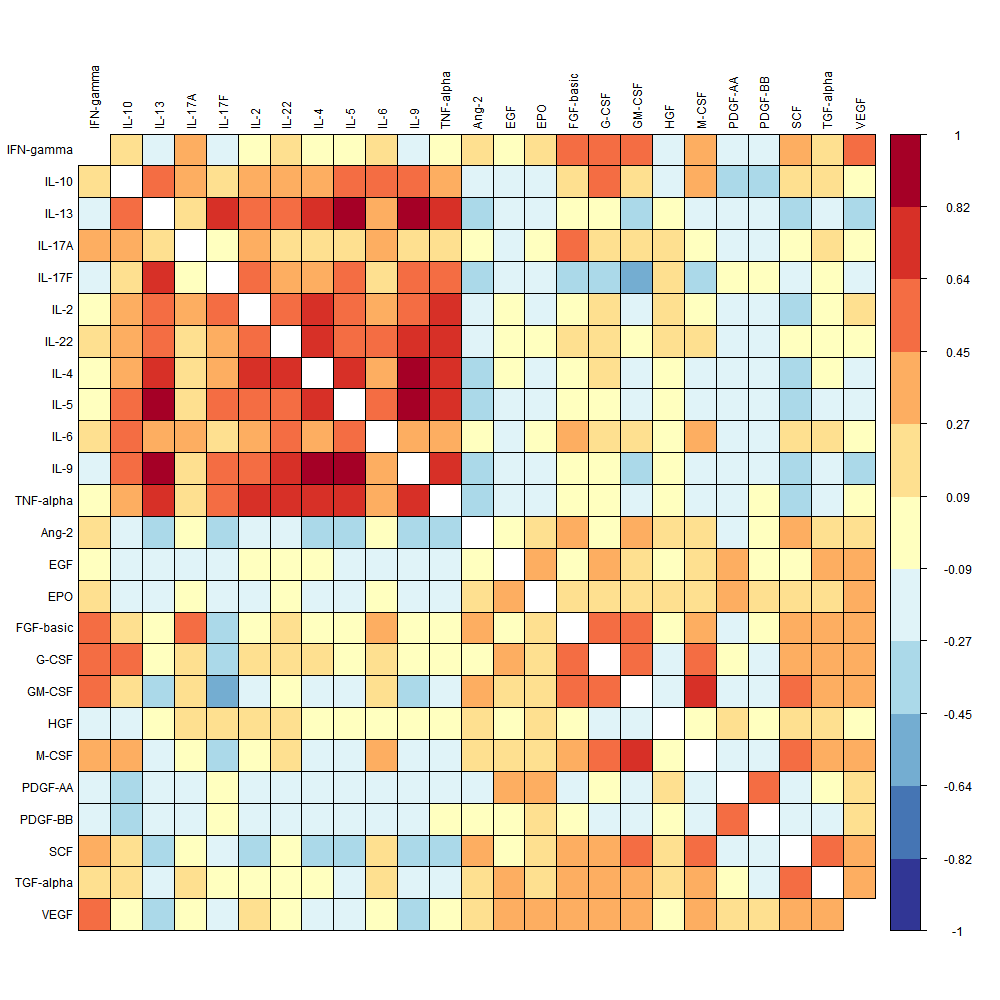

Supplement: Supplementary file 1 [file viruses-14-01613-s001.zip › Figure_S1.tiff]

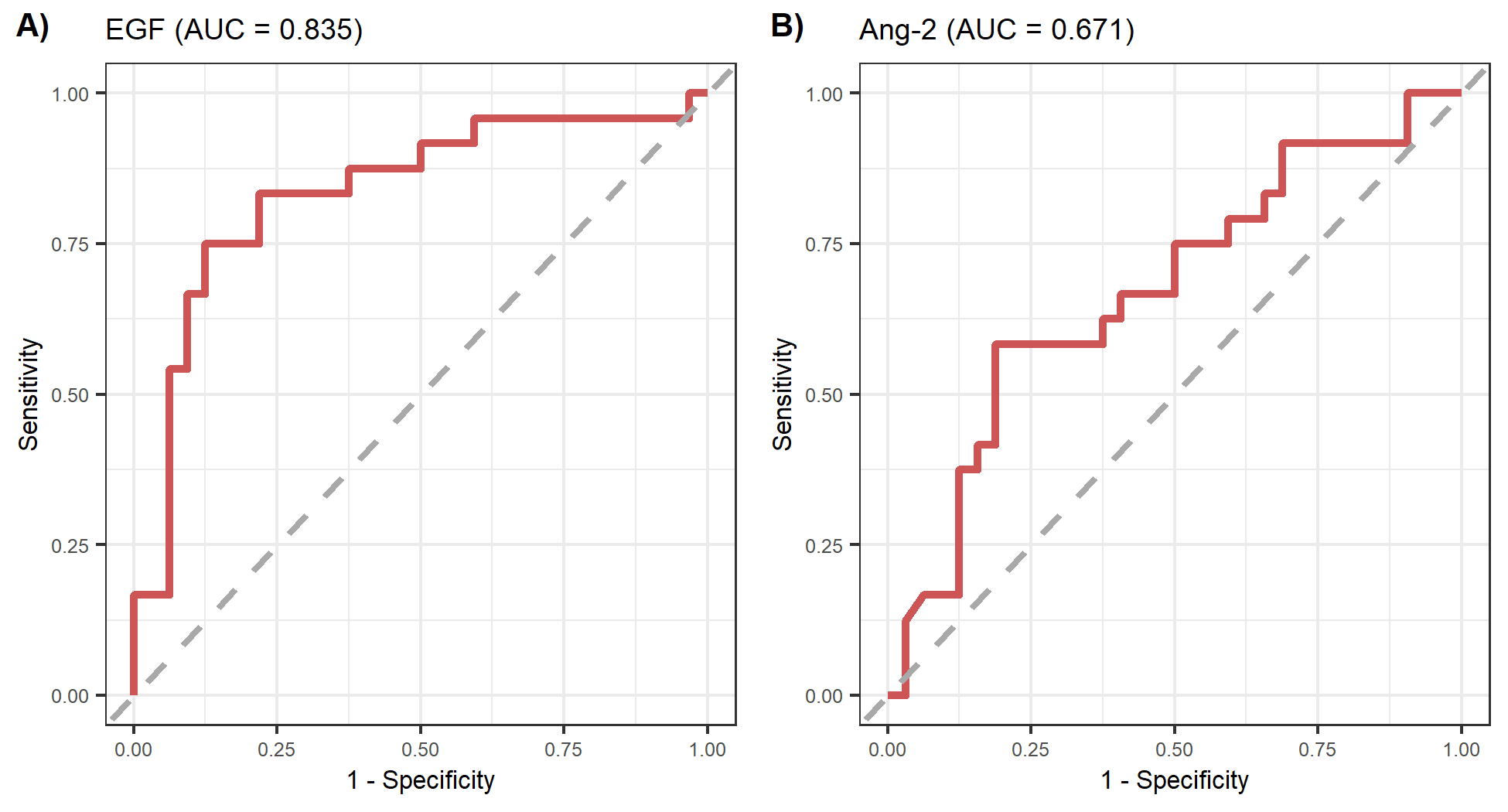

Supplement: Supplementary file 1 [file viruses-14-01613-s001.zip › Figure_S2.tiff]
